# Giant low-grade endometrial stromal sarcoma with extensive sex cord–like differentiation: a case report

**DOI:** 10.1097/RC9.0000000000000453

**Published:** 2026-06-25

**Authors:** Wafaa Almohammad Alhamdo, Abdullah Alfandi, Khalid Khattab, Ali Jumaa, Mohammad Adi, Majd Mohammad Al Ali

**Affiliations:** a Gynecology Department, Al-Nour Hospital, Idlib, Syria; b Faculty of Medicine, Al-Sham Private University, Damascus, Syria; c Faculty of Medicine, Damascus University, Damascus, Syria; d Faculty of Medicine, Latakia University, Latakia, Syria

**Keywords:** case report, endometrial stromal sarcoma, low-grade endometrial stromal sarcoma

## Abstract

**Background:**

A low-grade endometrial stromal sarcoma (LG-ESS) is a rare uterine malignancy that can resemble benign conditions like leiomyoma or adenomyosis. Diagnosis is challenging, particularly when sex cord–like differentiation is present, which can mimic other uterine or ovarian tumors.

**Case presentation:**

A 45-year-old woman presented with pelvic discomfort and heavy bleeding. Imaging suggested a 13-cm leiomyoma. She underwent a total hysterectomy with bilateral salpingo-oophorectomy. Histology showed classic LG-ESS features with extensive sex cord–like areas. Immunohistochemistry confirmed the presence of an LG-ESS (CD10+, ER+, inhibin+, and calretinin+).

**Discussion:**

An extensive sex cord–like differentiation in an LG-ESS is rare and may lead to misdiagnosis. Accurate identification requires immunostaining to distinguish from mimics. Prognosis remains favorable with complete surgical resection. Ovarian removal was chosen due to the patient’s age and tumor features.

**Conclusion:**

This case highlights a rare LG-ESS variant with unusual morphology, stressing the importance of histopathological evaluation and long-term follow-up to monitor for late recurrence.

## Introduction

Uterine sarcomas comprise a diverse group of rare malignant tumors arising from the uterine muscle and connective tissue. Uterine sarcomas represent about 2–5% of all uterine cancers, with an incidence of roughly 1–2 cases per 100 000 women. Endometrial stromal sarcomas make up around 10% of these sarcomas, accounting for only about 0.2% of all uterine malignancies. According to the latest WHO classification, these neoplasms are subdivided histopathologically into several types, including leiomyosarcoma, low-grade endometrial stromal sarcoma (LG-ESS), high-grade ESS, undifferentiated uterine sarcoma, adenosarcoma, rhabdomyosarcoma, and malignant perivascular epithelioid cell tumors^[^1^]^.

HIGHLIGHTS

- We report a giant (13 cm) low-grade endometrial stromal sarcoma (LG-ESS), an uncommon size for this typically indolent tumor.
- The tumor showed an extensive sex cord–like differentiation, a rare finding that complicates diagnosis by mimicking other uterine or ovarian neoplasms.
- Immunohistochemistry (CD10, ER, PR, inhibin, and calretinin) was essential to confirm the diagnosis and exclude mimics like UTROSCT.
- Despite its large size, the tumor retained classic low-grade features, supporting a complete surgical resection as an effective treatment.
- This case highlights the importance of long-term follow-ups, as LG-ESSs may recur many years after apparently curative surgery.

The LG-ESS is the second most common uterine mesenchymal tumor after leiomyosarcoma, with an annual incidence of about 0.19 per 100 000 women. ESSs represent 0.2% of uterine malignancies and 7–25% of uterine sarcomas. ESSs most commonly affect women in the peri- and postmenopausal periods, with a median age of approximately 46 years. An LG-ESS is characterized by an indolent course, slow-growing and estrogen receptor positivity. The LG-ESS usually presents at early stages and is confined to the uterus, manifesting as abnormal uterine bleeding, pelvic pain, or a pelvic mass. The main differential diagnoses include leiomyoma and endometrial polyp. The condition typically exhibits a mean recurrence time of approximately 6 years, with a 5-year overall survival rate of around 80%, indicating a relatively favorable long-term clinical prognosis^[^1,2^]^. This case report has been reported in line with the SCARE checklist^[^3^]^.

## Case presentation

A 45-year-old woman presented to the gynecology outpatient clinic with a several-month history of progressively worsening abnormal uterine bleeding associated with fatigue and exertional dyspnea. She reported regular menstrual cycles but noted marked menorrhagia over recent months. The family, medical, and surgical history was unremarkable.

**Figure 1.**
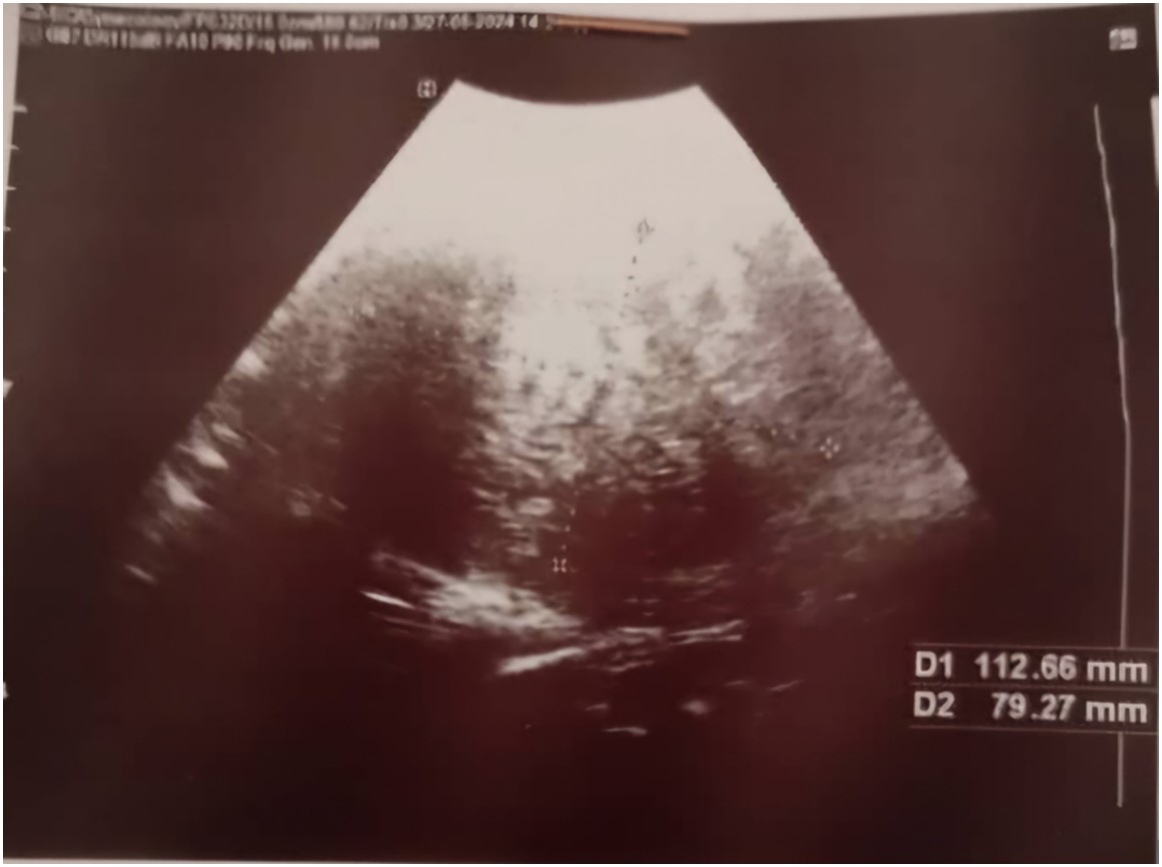
Transvaginal pelvic ultrasound showing a markedly enlarged uterus with a well-circumscribed, heterogeneous, hypoechoic mass located on the right uterine side, measuring approximately 8 × 11 cm. The endometrial stripe is displaced but not invaded. The mass was initially interpreted as a large intramural leiomyoma. Both ovaries appear normal in size and echotexture, with no evidence of masses or calcifications.

On general examination, the patient appeared pale, with signs of anemia. Abdominal examination revealed significant uterine enlargement, with the uterine fundus palpable above the level of the umbilicus, suggesting a mass effect. No adnexal masses were palpated. Initial laboratory evaluation revealed severe normocytic, normochromic anemia, with a hemoglobin level of 5.0 g/dl. Iron studies were consistent with iron deficiency anemia secondary to chronic blood loss, and the reticulocyte count was mildly elevated, supporting ongoing hemorrhage. A transvaginal ultrasound and subsequent pelvic MRI revealed a markedly enlarged uterus, measuring approximately 18 × 15 × 11 cm, containing a large right-sided uterine mass measuring approximately 8 × 11 cm (Fig. 1). The imaging findings were initially interpreted as suggestive of a large leiomyoma, with no involvement of the endometrial cavity. Both ovaries were visualized and appeared normal on imaging, with no evidence of calcifications or masses.

Given the severity of the patient’s anemia and the need to establish a definitive diagnosis, an initial exploratory endometrial curettage was performed. However, the pathologist deemed the specimen non-diagnostic due to the presence of scattered adipocytic cells, and a repeat sampling or definitive surgical excision was recommended. Consequently, although the sample remained insufficient to render a conclusive histopathological diagnosis, a second curettage was carried out, which revealed atypical cellular features suspicious for malignancy.

The clinical suspicion of an underlying malignant process was heightened by the patient’s refractory anemia despite multiple transfusions and the rapid progression in uterine size. In light of these findings, and in the absence of a definitive preoperative diagnosis, the multidisciplinary team proceeded with definitive surgical intervention. The patient underwent an elective total abdominal hysterectomy with bilateral salpingo-oophorectomy. Intraoperatively, the uterus appeared markedly enlarged and irregular, harboring a 13-cm mass infiltrating the myometrial wall. There was no evidence of serosal involvement, peritoneal implants, lymphadenopathy, or pelvic adhesions.

**Figure 2.**
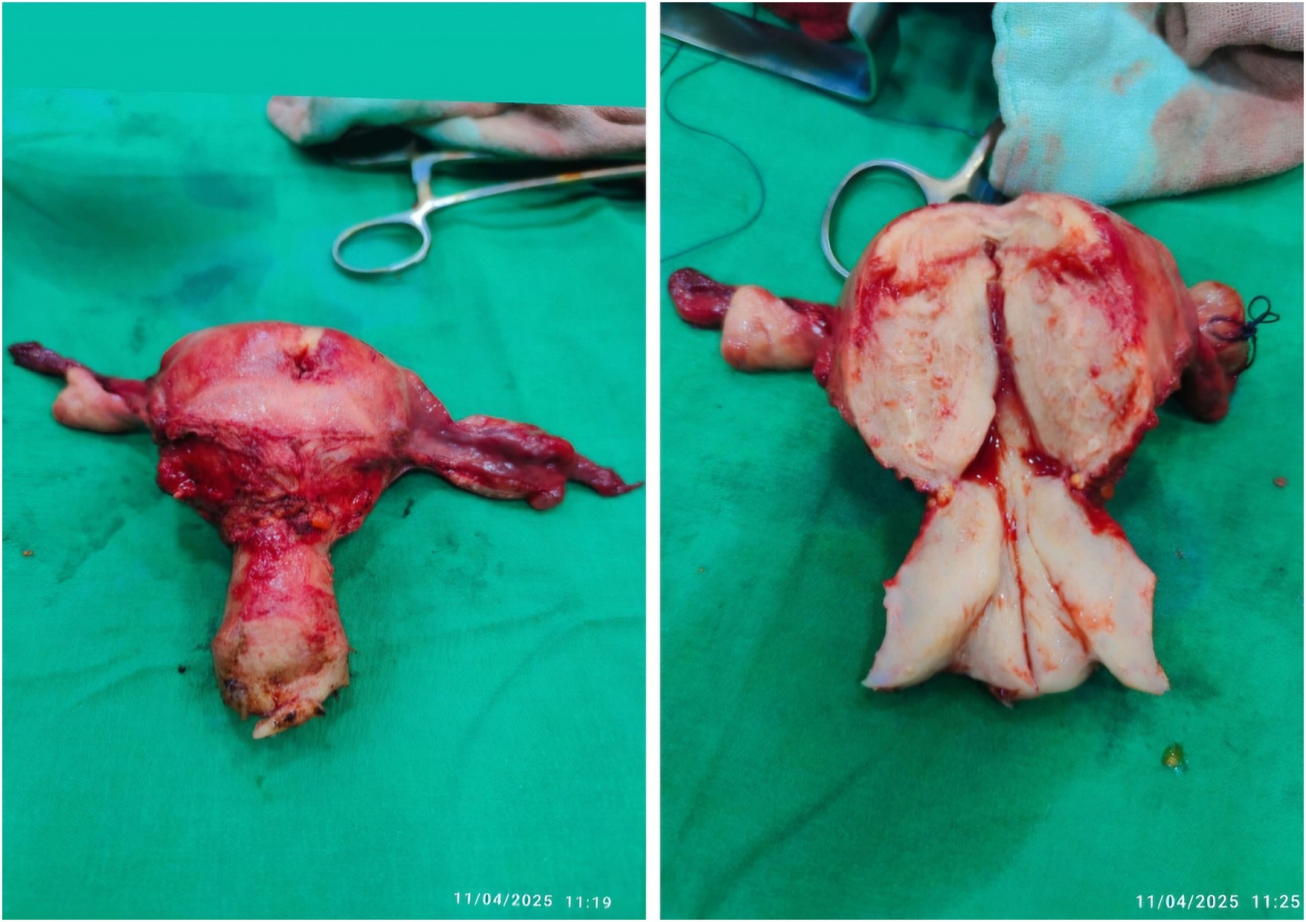
Gross photograph of the resected uterus showing an enlarged uterine corpus (18 × 15 × 11 cm) with a tan-white infiltrative mass measuring 13 × 10 cm, replacing most of the myometrial tissue. The mass appears poorly circumscribed and diffusely invasive. The cervix is grossly unremarkable. The right ovary shows small cystic structures, while the left ovary and both fallopian tubes appear normal.

Gross pathological examination of the resected specimen revealed an enlarged uterus measuring 18 × 15 × 11 cm with a diffusely infiltrative white-tan mass measuring 13 × 10 cm that replaced the majority of the myometrium. The cervix measured 4 × 4 × 3 cm and appeared unremarkable on gross examination. The right ovary measured 4 × 4 × 3 cm and contained small cystic formations, the largest measuring 1.5 cm, while the left ovary measured 4 × 3 × 2 cm. Both fallopian tubes appeared grossly unremarkable (Fig. 2).

Microscopic examination revealed a tumoral mass infiltrating diffusely into the myometrium, composed of irregular cellular islands forming a permeative, tongue-like pattern of myometrial invasion with frequent vascular invasion. The tumor cells were monotonous and oval-shaped, with minimal cytologic atypia, vesicular chromatin, and scant cytoplasm. Mitotic activity was low (<5/10 high-power fields) but a marked necrosis was observed. Tumor cells exhibited a characteristic whirling pattern around delicate arteriolar-type vessels, reminiscent of proliferative-phase endometrial stroma. Areas of collagen plaques and foamy histiocytes were also noted. Minimal smooth muscle differentiation was observed, with a predominance of sex cord-like features. Immunohistochemistry demonstrated strong positivity for CD10, estrogen, and progesterone receptors, supporting the diagnosis of an LG-ESS. Sections from both ovaries and fallopian tubes showed no evidence of malignancy. The cervix displayed hyperplastic squamous epithelium with underlying mucous endocervical glands, chronic fibrous and edematous tissue, and multiple Nabothian cysts, without evidence of tumor extension. The final diagnosis was an LG-ESS of the uterine corpus, with free surgical margins and no involvement of the cervix or extrauterine structures. Bilateral ovaries and fallopian tubes were free of tumor. Histopathological evaluation confirmed the confinement of the LG-ESS to the uterine corpus, measuring 13 cm with no cervical, adnexal, or extrauterine involvement. According to the TNM classification, the tumor was staged as pT1b pN0 pM0, corresponding to FIGO stage IB.

The postoperative course was uneventful. In view of the tumor’s low-grade histology and the absence of extrauterine disease, adjuvant radiotherapy or chemotherapy was not administered. However, given the hormone receptor positivity of the tumor and the known risk of late recurrence of the LG-ESS, an adjuvant hormonal therapy was initiated. The patient was started on an oral administration of letrozole (2.5 mg) once every day, with a planned treatment duration of at least 5 years, contingent on tolerance and absence of disease recurrence. The patient was enrolled in a structured surveillance program consisting of pelvic examination and imaging every 6 months for the first 2 years, followed by annual assessments thereafter.

## Discussion

Uterine sarcomas and benign leiomyomas often present with overlapping nonspecific clinical manifestations, such as abnormal vaginal bleeding, pelvic pain, and palpable abdominal masses. No specific clinical or radiological criteria reliably differentiate these two entities. However, rapid growth of a presumed fibroid, particularly in peri- or postmenopausal women, should prompt suspicion of sarcoma and warrant further evaluation for malignancy. The LG-ESS is a rare uterine malignancy often mistaken preoperatively for benign leiomyomas, especially when presenting with heavy menstrual bleeding and uterine enlargement, as seen in the present case^[^1,2,4^]^. Imaging modalities like ultrasound, CT, and MRI lack specificity for diagnosing LG-ESSs. The definitive diagnosis of LG-ESSs was established postoperatively based on histopathology. Misdiagnosis occurs in roughly 20% of LG-ESS cases^[^5,6^]^.

The ESS is staged using the FIGO and TNM classification systems, which help determine prognosis and guide management. Stage I disease is confined to the uterus, with Stage IA and Stage IB limited to tumors of ≤5 cm and >5 cm in size, respectively. Stage II indicates pelvic extension, involving either the adnexa (IIA) or other pelvic tissues (IIB). Stage III denotes tumor infiltration into the abdomen (IIIA for a single site and IIIB for multiple sites) or lymph node metastasis (IIIC). Stage IV involves either local invasion of the bladder or rectum (IVA) or distant metastases (IVB). In the present case, the tumor was confined to the uterus and measured 13 × 10 cm in its largest dimension, placing it in Stage IB (FIGO IB/TNM T1b)^[^1^]^. This highlights the low-grade nature of the tumor but also emphasizes the importance of accurate staging and thorough histopathological evaluation to guide postoperative management.

LG-ESSs generally consist of uniform spindle or oval cells with low mitotic activity and mild atypia. Sex cord–like areas – appearing as trabeculae, cords, or tubules – are typically focal and limited in extent. These regions mimic ovarian sex cord tumors but usually comprise a minor component of the neoplasm. Extensive sex cord–like differentiation, as seen in the present case, is uncommon and can create diagnostic challenges by mimicking other neoplasms such as UTROSCT^[^4,5^]^. An LG-ESS with a sex cord–like differentiation poses notable diagnostic pitfalls. Clinically and radiologically, the LG-ESS mimics benign fibroids – ultrasound, CT, and MRI lack specific features to distinguish it. Accordingly, preoperative diagnoses are often uterine leiomyoma or adenomyosis, as in the present case. Sampling by curettage or needle biopsy frequently yields insufficient or misleading tissue (e.g., smooth muscle–like cells in degenerated areas); thus, the tumor may be missed. Extensive sex cord–like components further complicate the picture by resembling ovarian sex-cord tumors or UTROSCT; a broad immunohistochemical panel is required to sort this out. In practice, thorough histopathology is the gold standard. LG-ESS cells are usually diffusely CD10 and ER/PR positive, while any sex cord–like region may express inhibin or calretinin. Management also has special considerations. As LG-ESSs are estrogen-driven, many experts perform bilateral oophorectomy even in premenopausal patients to eliminate hormonal stimulation. Conversely, routine lymphadenectomy has not shown survival benefit and is generally omitted. In summary, the rarity of a sex cord–rich LG-ESS means that each case must be managed with a high index of suspicion, meticulous pathology review, and individualized planning^[^4,5,7^]^.

LG-ESSs are typically indolent neoplasms that present at smaller sizes. In a recent review, Salehi *et al* report that LG-ESS tumors range widely in size (1–25 cm) with a mean of about 8–11 cm, underscoring that a 13-cm lesion is at the high end of the usual spectrum. Indeed, giant LG-ESSs have rarely been documented; for example, Gnangnon *et al* had described an LG-ESS measuring 32 × 25 × 13 cm – yet, histologically it exhibited only “discreet cellular atypia and low mitotic activity (<5 per 10 HPF).” These reports illustrate that even very large uterine masses can retain a bland, low-grade histology^[^6,8^]^.

This paradoxical combination – a large bulk but minimal cytologic atypia – has important diagnostic implications. Such a mass may initially mimic a benign leiomyoma on imaging and intraoperative inspection, and bland cytology on limited sampling can be misleading. Therefore, the recognition of the fact that LG-ESSs can achieve large sizes despite a low mitotic index is essential. In practice, any large uterine tumor should be carefully sampled and evaluated (e.g., with CD10 immunostaining and attention to infiltrative growth) to rule out an LG-ESS, even when the atypia is minimal. In the present case, the 13-cm size combined with low-grade features is unusual but in line with these reports, reminding clinicians that a large size alone does not exclude an indolent ESS^[^8^]^.

LG-ESSs share several risk factors with other uterine malignancies. These include endometriosis, heightened exposure to estrogen, obesity, older age, prior pelvic radiation, tamoxifen use, and genetic predispositions such as the Lynch syndrome. A national cohort study demonstrated that individuals with endometriosis have a significantly increased risk of developing uterine sarcomas, attributed to chronic estrogen-driven inflammation. In the index case, the patient presented none of these established risk factors^[^9,10^]^.

The cornerstone of LG-ESS management is surgery by total hysterectomy with bilateral salpingo-oophorectomy. In practice, this is curative for early-stage LG-ESSs. As LG-ESS is usually ER/PR-positive, most experts advise adjuvant endocrine therapy to reduce relapse risk. Retrospective series show dramatically lower recurrence with progestins or aromatase inhibitors: one series reported stage I recurrences of 70% without hormonal therapy versus only ~14% with an aromatase inhibitor and ~8% with progestins. The Spanish uterine-sarcoma guidelines summarize these data (e.g., relapse risk falling from ~67% to ~31% with megestrol) and “recommend considering” adjuvant progestins or aromatase inhibitors for LG-ESSs. By contrast, if the patient is premenopausal, removing the ovaries is especially important to eliminate estrogen drive. When hormonal therapy is prescribed it is usually continued for years; therefore, these patients should be monitored for side effects, such as changes in bone density associated with an aromatase inhibitor therapy^[^1,11^]^.

Given the high hormone receptor expression, adjuvant endocrine therapy is the cornerstone for preventing recurrence of LG-ESSs. Chemotherapy is usually avoided (LG-ESSs have very low mitotic rates and have a poor chemo-response) and radiotherapy offers local control only. Current guidelines (NCCN, ESGO, etc.) reflect this; they advise antiestrogen hormone therapy (e.g., progestins or aromatase inhibitors) for advanced (stage II+) LG-ESSs, with observation or risk-adapted therapy in low-risk stage I. Importantly, even early-stage patients benefit from adjuvant hormones. For example, one multi-institutional series found that 70% of stage I^[^12^]^ patients had recurrence without any hormonal treatment, versus only ~14% with an aromatase inhibitor and 7–8% with progestins. Thus, many clinicians now recommend prolonged adjuvant endocrine therapy after surgery for LG-ESSs to reduce relapse risk. In the present case, the patient’s tumor was strongly ER/PR-positive; so, we initiated letrozole for this reason. In contrast, neither chemotherapy nor routine radiation is clearly indicated for low-grade diseases. Overall, an adjuvant therapy (especially hormonal) is employed when any high-risk features are present or for advanced-stage diseases, to leverage the tumor’s hormone-dependence and improve long-term outcomes^[^11,12^]^.

A long-term follow-up is required due to the indolent clinical course and well-documented risk of the late recurrence of LG-ESSs. Current guidelines recommend regular pelvic examination and imaging surveillance following primary treatment. Standard practice includes cross-sectional imaging of the chest, abdomen, and pelvis at defined intervals during the early postoperative years, followed by less frequent but continued long-term surveillance. This approach is justified by reports demonstrating that recurrences, most commonly pelvic but occasionally pulmonary, may occur many years after initial surgical management. Patients should be educated regarding symptoms suggestive of relapse, such as abnormal vaginal bleeding or pelvic pain, and advised to seek prompt medical evaluation should these occur. Collectively, a complete surgical resection, the consideration of prolonged hormonal therapy, and a diligent long-term follow-up constitute current best practice for the management of stage I LG-ESSs^[^1^]^.

Low-grade ESSs have an overall favorable prognosis, but long-term survival depends on controlling late recurrences and distant spread. In practice, the leading causes of death are metastatic recurrences rather than the primary uterine tumor. Published series report that roughly 15–20% of LG-ESS patients eventually die of their disease^[^12^]^. The most frequent distant site is the lung – about 7–28% of cases with extrapelvic metastases involve pulmonary lesions. Other documented metastatic sites include liver and bone^[^13,14^]^. Rarely, LG-ESSs can invade large vessels or even reach the heart. Thus, preventing and promptly detecting a distant recurrence (especially lung metastasis) is critical, as these disseminated lesions account for most of the LG-ESS–related mortalities^[^7^]^.

A PubMed search of English-language, medical literature was performed using the keywords *low-grade endometrial stromal sarcoma, endometrial stromal sarcoma, sex cord differentiation, sex cord–like*, and *case report*. Original case reports and small case series describing LG-ESSs with sex cord–like differentiation were included, while reviews and unrelated entities were excluded. Clinicopathological and immunohistochemical data were extracted from eligible studies. Using this strategy, nine studies were identified, comprising seven case reports and two small case series published between 1997 and 2025^[^5,6,8,15–20^]^ (Table 1).

**Table 1:** Published cases of low-grade endometrial stromal sarcoma with sex cord–like differentiation.

Author (year)	No. of cases	Age (years)	Tumor size (cm)	Extent of sex cord–like differentiation	Key immunohistochemistry	Stage/Spread	Remarks
Fukunaga *et al* (1997)^[^16^]^	2	39, 42	7.0; 7.5	Predominant trabecular/cord-like pattern	CD10 +, ER/PR +	Local; 1 metastatic	First detailed description; diagnostic pitfall due to dominant sex cord pattern
Pang *et al* (1998)^[^20^]^	2	Postmenopausal patients	Not large	Sex-cord-like areas described (focal to more conspicuous)	—	Tamoxifen association reported in some cases	Hysterectomy
Richmond *et al* (2016)^[^5^]^	1	50	NR	Extensive, with heterologous elements	CD10+, ER/PR+, inhibin+	Uterus confined	Emphasized distinction from UTROSCT; extensive sampling required
Zhu *et al* (2019)^[^17^]^	1	47	NR	Mixed sex cord–like and smooth muscle	CD10+, ER/PR+	Recurrent/metastatic	Rare combination; aggressive clinical course despite low-grade histology
Sinha *et al* (2021)^[^15^]^	1	47	~10	Prominent sex cord differentiation	CD10+, ER/PR+, inhibin+	Stage I	Nearly identical diagnostic scenario; mimicked leiomyoma
Gnangnon *et al* (2022)^[^8^]^	1	49	32 × 25 × 13	Not specified (a classic LG-ESS)	CD10+, ER/PR+	Metastatic	Demonstrates that very large size does not preclude low-grade biology
Salehi *et al* (2023)^[^6^]^	1	52	NR	Not specified	CD10+, ER/PR+	Ovarian metastasis	Illustrates extrauterine spread; no sex cord focus reported
Al-Maghrabi *et al* (2024)^[^18^]^	1	60	NR	Sex cord–like differentiation in metastasis	CD10+, ER/PR+, inhibin+	IVC metastasis	Unique: sex cord features appeared only in metastatic lesion
Zhao *et al* (2025)^[^19^]^	1	44	NR	Focal sex cord-like areas	CD10+, ER/PR+	Stage I	Initially misdiagnosed as fibroids
Present case	1	45	13	*Extensive, predominant component*	CD10+, ER/PR+, inhibin+, calretinin+	FIGO IB	Giant LG-ESS with marked sex cord differentiation

A sex cord–like differentiation in an LG-ESS is exceedingly rare as ESSs with sex cord elements occur only in 0.25% of cases^[^11^]^. Most published information comes from isolated case reports and small series. In each instance, thorough histopathological examination and immunohistochemistry (e.g., CD10, inhibin, and hormone receptors) were required to recognize the diagnosis. No large cohort studies exist; treatment in all reported cases was surgical, with hormone therapy considered given the ER/PR positivity of the LG-ESSs. The reported studies are summarized in Table 1^[^5,6,8,15–20^]^.

Similar to the cases described by Richmond *et al* and Fukunaga *et al*, the tumor shows a prominent sex cord–like component that can closely mimic ovarian sex cord–stromal tumors, underscoring the diagnostic difficulty encountered in the preoperative and histopathological assessment^[^5,16^]^. In contrast to reports describing metastatic disease or the presence of additional heterologous elements, such as a smooth-muscle differentiation, the tumor in the current case was confined to the uterus without evidence of extrauterine spread at diagnosis, corresponding more closely to early-stage disease presentations documented in the literature. Notably, the application of a broad immunohistochemical panel – including CD10, estrogen receptor (ER), progesterone receptor (PR), inhibin, and calretinin – is consistent with recommended diagnostic approaches for differentiating LG-ESSs with sex cord–like elements from uterine tumors resembling ovarian sex cord tumors (UTROSCTs) and related entities. Furthermore, the considerable tumor size observed in this case contributes to the expanding spectrum of reported tumor dimensions in LG-ESSs with sex cord–like differentiation, complementing prior reports in which tumor size varied substantially and, in some metastatic cases, exceeded 25 cm^[^5,6,8,15–20^]^.

## Conclusion

We report an unusual giant (13 cm) LG-ESS with an extensive sex cord–like differentiation. The patient recovered well postoperatively. This case highlights the need to consider LG-ESSs in the differential of large uterine masses and the importance of a thorough pathological assessment. A long-term individualized follow-up is crucial, as LG-ESSs can recur many years later. Tailoring surgical decisions and surveillance strategies to each patient’s risk profile remains essential for optimal outcomes.

## Data Availability

All data generated or analyzed during this study are included in this published article.

## Artificial intelligence (AI) declaration

AI (ChatGPT, GPT-5, and OpenAI) was used only for reviewing, polishing, and correcting the language of the manuscript to improve clarity and readability. No generative content, scientific interpretation, or data analysis was produced by AI. The AI tool was used on the manuscript text only, with no patient data or sensitive information provided. All AI-assisted edits were reviewed, verified, and approved by the authors, who take full responsibility for the integrity and accuracy of the manuscript.
